# Impact of Cannabis Use on Treatment Outcomes among Adults Receiving Cognitive-Behavioral Treatment for PTSD and Substance Use Disorders

**DOI:** 10.3390/jcm6020014

**Published:** 2017-02-07

**Authors:** Lesia M. Ruglass, Alina Shevorykin, Vanja Radoncic, Kathryn M. Z. Smith, Philip H. Smith, Isaac R. Galatzer-Levy, Santiago Papini, Denise A. Hien

**Affiliations:** 1 Department of Psychology, The City College of New York, CUNY, 160 Convent Avenue, NAC Building, Rm 7/120, New York, NY 10031, USA; 2 Department of Psychology, Pace University, 861 Bedford Road, Pleasantville, NY 10570, USA; alina.shevorykin@gmail.com; 3 Gordon F. Derner Institute for Advanced Psychological Studies, Adelphi University, IAPS, Hy Weinberg Center, Room 306, Garden City, NY 11530-0701, USA; vanyaradoncic@gmail.com; 4 Division on Substance Use Disorders, Department of Psychiatry, Columbia University Medical Center/New York State Psychiatric Institute, 1051 Riverside Drive, Box 66, New York, NY 10032, USA; k.m.zumberg@gmail.com; 5 Sophie Davis School of Biomedical Education, The City College of New York, 160 Convent Avenue, New York, NY 10031, USA; psmith@med.cuny.edu; 6 Department of Psychiatry, NYU School of Medicine, 1 Park Avenue, New York, NY 10016, USA; isaac.galatzer-levy@nyumc.org; 7 Department of Psychology and Institute for Mental Health Research, University of Texas, Austin, 108 E. Dean Keeton Street, Austin, TX 78712, USA; papinisan@gmail.com; 8 Gordon F. Derner Institute for Advanced Psychological Studies, Adelphi University & Department of Psychiatry, Columbia University College of Physicians and Surgeons, Hy Weinberg Center, Room 306, Garden City, NY 11530-0701, USA; dr.denise.hien@gmail.com

**Keywords:** trauma, PTSD, cannabis, substance use disorder, treatment outcomes

## Abstract

Background: Research has demonstrated a strong link between trauma, posttraumatic stress disorder (PTSD) and substance use disorders (SUDs) in general and cannabis use disorders in particular. Yet, few studies have examined the impact of cannabis use on treatment outcomes for individuals with co-occurring PTSD and SUDs. Methods: Participants were 136 individuals who received cognitive-behavioral therapies for co-occurring PTSD and SUD. Multivariate regressions were utilized to examine the associations between baseline cannabis use and end-of-treatment outcomes. Multilevel linear growth models were fit to the data to examine the cross-lagged associations between weekly cannabis use and weekly PTSD symptom severity and primary substance use during treatment. Results: There were no significant positive nor negative associations between baseline cannabis use and end-of-treatment PTSD symptom severity and days of primary substance use. Cross-lagged models revealed that as cannabis use increased, subsequent primary substance use decreased and vice versa. Moreover, results revealed a crossover lagged effect, whereby higher cannabis use was associated with greater PTSD symptom severity early in treatment, but lower weekly PTSD symptom severity later in treatment. Conclusion: Cannabis use was not associated with adverse outcomes in end-of-treatment PTSD and primary substance use, suggesting independent pathways of change. The theoretical and clinical implications of the reciprocal associations between weekly cannabis use and subsequent PTSD and primary substance use symptoms during treatment are discussed.

## 1. Introduction

Decades of research have demonstrated a strong link between trauma, PTSD and Substance Use Disorders (SUDs) in general [1,2,3,4] and cannabis use disorders in particular [5]. In one large sample of veterans seeking treatment for PTSD, 14.6% of participants reported using cannabis in the previous six months [6], and 14% of individuals with lifetime PTSD reported past-year cannabis use [7]. In a nationally representative adult sample, lifetime trauma was associated with greater likelihood of lifetime cannabis use, and results showed a graduated relationship between the co-occurrence of the two, with less severe cannabis use being related to less severe trauma exposure [8]. A number of etiological models have been proposed to explain the co-occurrence of PTSD and substance use, including the “self-medication” hypothesis, “high risk” hypothesis, “susceptibility” hypothesis and “shared vulnerability” models [9,10,11,12,13,14]. The self-medication hypothesis posits that individuals with PTSD use substances as a way to manage painful affect states [15,16]. Indeed, a substantial portion of individuals with anxiety and/or PTSD reports symptom relief as a goal of their substance use [17,18,19]. While the self-medication hypothesis has received the most clinical attention and empirical support, several other hypotheses may account for the complex relationships between PTSD and SUD. The high risk hypothesis posits that a substance-using lifestyle (e.g., illegal procurement of drugs) places the individual at high risk for exposure to traumatic events and subsequent development of PTSD [12,14]. Relatedly, the susceptibility hypothesis proposes that chronic substance use impairs one’s neurobiological system, which enhances the likelihood of developing PTSD after trauma exposure [9,11]. Shared vulnerability models implicate common cognitive, affective and neurobiological factors (e.g., attentional bias, emotion regulation difficulties and dysfunction in the hypothalamic-pituitary-adrenal (HPA) axis) in the development of both PTSD and SUD and their associations [20]. For example, previous research shows that adults with either PTSD or SUDs exhibit deficits in emotion processing and self-regulation [21,22].

Cannabis is often used concurrently with other substances, with individuals who used cannabis in the previous six months being more likely than those who do not use cannabis to smoke cigarettes (33.9% vs. 61.9%), engage in hazardous alcohol use (8.89% vs. 11.54%) and other drug use (4.1% vs. 23.8%) [6]. In a sample of adolescents, ongoing regular cannabis use was found to predict the maintenance of other drug use and to be associated with reduced rates of cessation for high-risk alcohol use and use of all other substances, with the exception of cocaine [23]. The latter exception was due to lower prevalence and sporadic use of cocaine compared to other drugs in this sample. A recent prospective study of 34,653 participants found that use of cannabis at baseline was a significant predictor of the development of a SUD three years later, even after adjusting for other sociodemographic factors known to contribute to SUDs [24]. Furthermore, use of multiple substances, often termed polysubstance use, has been associated with elevated risk of psychiatric and physical health problems, an increased risk of dependence and overdose and poor treatment outcomes [25]. Interestingly, Swift et al. found that although overall as cannabis users age, prevalence of cannabis use declines, a greater proportion of users become regular users, and the prevalence of other illicit drug use increases and progresses [23].

Given the widespread utilization of cannabis among those with PTSD, paired with reductions in the perception of the harmfulness of cannabis, investigations have now begun to explore whether the utilization of cannabis prior to, during or after treatment is associated with beneficial or adverse treatment outcomes among those with co-occurring psychiatric and other substance use disorders. Several studies suggest that cannabis use is associated with PTSD symptom reduction and improved coping [26,27]. In a study of coping strategies among veterans experiencing PTSD, a subgroup of participants reported preferring cannabis to other types of substances and experiencing benefits from cannabis use, including reductions in anxiety and depression and prevention of intrusive thoughts and memories [28]. A recent literature review on military veterans with PTSD identified several small studies showing that cannabis and cannabinoid use were associated with reductions in PTSD symptoms [26]. Evidence suggests the psychoactive properties of cannabis may be helpful in reducing anxiety, extinguishing fears and aversive memories, improving depression and enhancing sleep [28,29]. However, chronic cannabis use has been shown to impair fear extinction in preclinical models relevant to PTSD [30,31,32].

The benefits of cannabis use with respect to other drugs of abuse are less clear. Several studies have reported no association between cannabis use and adverse treatment outcomes among opioid- and cocaine-dependent patients [33,34,35,36]. For example, a study of opioid-dependent youth who received psychosocial treatments plus buprenorphine-naloxone found that cannabis use prior to treatment onset and concurrent cannabis use were not associated with opioid use over 12 weeks of treatment [35]. The authors theorized that cannabis may not have had an impact on outcomes because the buprenorphine-naloxone treatment may have adequately addressed many of the symptoms that individuals are likely to use cannabis to relieve (e.g., dysphoria and opioid withdrawal symptoms) [35]. Likewise, a retrospective chart review of individuals during the early phase of outpatient treatment found that cannabis use was associated with fewer days of opioid use and did not negatively impact the methadone induction process during methadone maintenance treatment [36]. Similar results have also been found among cocaine-dependent samples. In a sample of cocaine-dependent individuals seeking treatment, no significant adverse relationships were found between baseline and during treatment cannabis use and treatment retention and cocaine abstinence, suggesting that cannabis use and cocaine use were functionally independent [37]. Given the lack of impairment or adverse outcomes associated with cannabis use, these findings provide support for a harm reduction approach with regard to concurrent cannabis use.

However, other studies suggest that cannabis use either before entering or after being discharged from treatment may be associated with adverse treatment outcomes among opioid and cocaine-dependent patients [38,39,40]. For example, a prospective study of heroin-abstinent patients found that cannabis use was a significant predictor of heroin use resumption [40]. A secondary analysis of two completed clinical trials of treatment for cocaine dependence found an effect of baseline marijuana use on response to treatment for cocaine dependence; specifically, more days of marijuana use at baseline predicted lower treatment effectiveness (cocaine-negative urines) among those receiving levodopa/carbidopa, but not in the placebo condition [39]. The authors speculated that higher baseline marijuana use may be a proxy for cocaine severity. The counter argument that concurrent marijuana use can lead to negative outcomes on other substances proposes that cannabis use may activate the reward systems in the brain associated with alcohol, opioids and cocaine, leading to use or reinstatement of these other substances. These studies further suggest that cannabis use may not be as harmless as perceived and, thus, clinicians should actively assess for and incorporate cannabis use treatment in their client’s aftercare plan.

Despite mixed findings, the majority of studies reported were conducted with predominantly young, male, Caucasian or veteran samples receiving primarily medication-assisted treatments; thus, it remains unclear whether the findings are generalizable to other subgroups of populations. Moreover, none of the studies to date have examined the impact of cannabis use on treatment outcomes in a dually-diagnosed sample with co-occurring PTSD and substance use disorders. Additionally, very few studies have examined the effect of cannabis use on PTSD or primary substance use severity during treatment. The present study thus sought to extend prior findings by examining whether cannabis use at baseline and weekly during treatment had an impact on in-treatment and end-of-treatment PTSD symptom severity and primary substance use among a treatment-seeking dually-diagnosed population. Given the extant literature on the link between cannabis use and PTSD symptoms, we hypothesized that baseline and in-treatment cannabis use would be significantly associated with in-treatment and end-of-treatment PTSD symptom severity. Given the mixed findings in the literature on the impact of cannabis use on other substance use outcomes and the limited data on the effects of weekly cannabis use on in-treatment SUD symptomatology, we examined these associations in an exploratory fashion.

## 2. Experimental Section

### 2.1. Participants

Data for these analyses were derived from two recently-completed clinical trials that tested combined (behavioral and medication) and integrated cognitive-behavioral treatments for co-occurring PTSD and substance use disorders. Trial 1 compared Seeking Safety + Sertraline (SS + S) to Seeking Safety + Placebo (SS + P; see Hien et al. [41] for complete details on the procedures). Trial 2 compared Concurrent treatment with Prolonged Exposure therapy (COPE), Relapse Prevention Therapy (RPT) and an Active Monitoring Control Group (AMCG; see Ruglass et al. [42] for complete details on the procedures). Participants were randomly assigned to each treatment condition. Ethical approval was obtained for each of the clinical trials (Clinical Trials Registration: clinicaltrials.gov Identifier: NCT00262223 and NCT01365247) from which these data were drawn. All participants signed written informed consents approved by the institutional review board. Only participants who received active cognitive-behavioral treatments were included in these analyses. Participants who were randomized to the AMCG were excluded because they did not receive a cognitive-behavioral treatment intervention. We also excluded participants whose primary substance use disorder was cannabis use disorder (*n* = 9), since our primary question pertained to the impact of cannabis use on other primary substance use disorders. Of the 136 participants included in this analysis (out of 179), 32 participants used cannabis in the seven days prior to baseline and were included in the cannabis use group. The mean age of cannabis users was 41.63 years (SD = 9.38); approximately 66% were male; and average years of education were 13.08 (SD = 1.08). Nonusers had a mean age of 44 years old (SD = 9.18); approximately 42% were males; and they completed 13.28 years of education (SD = 2.52), on average. There were no significant differences in age (*t* = 1.273, *p* = 0.205), race/ethnicity distributions (*χ*^2^ = 5.12, *p* = 0.163), level of education (*t* = 0.426, *p* = 0.671) and level of employment (*χ*^2^ = 5.12, *p* = 0.402) between the cannabis users and nonusers. The cannabis user group was composed of significantly more males than nonusers (*χ*^2^ = 5.33, *p* = 0.021) and was more likely to have a cocaine use disorder than nonusers (*χ*^2^ = 4.686, *p* = 0.030). Nonusers were more like to have an alcohol use disorder compared to cannabis users (*χ*^2^ = 7.847, *p* = 0.005). See Table 1 for a summary of participants’ characteristics by cannabis use.

### 2.2. Cognitive-Behavioral Interventions

Trial 1: Seeking Safety (SS) [43] is a manualized 12-week intervention, which applies cognitive-behavioral strategies to the goals of attaining abstinence from substances and decreasing PTSD. It was delivered in a 12-session, 60-min weekly individual format by four experienced (Ph.D. or licensed clinical social worker (LCSW) level) research therapists who underwent rigorous training in the Seeking Safety protocol. Participants had up to 14 weeks to complete all 12 sessions. The content of each session was structured to provide a theme relevant to both substance use disorders and PTSD, and a specific cognitive-behavioral therapy (CBT) skill to learn. Medication: Matching capsules contained sertraline or placebo, as well as riboflavin to assess medication adherence. Compliance was also monitored by pill count. Participants receiving sertraline started on 50 mg daily and titrated up to 200 mg daily over a 2-week period. Participants continued on their full sertraline dose until the end of the trial and were tapered after unblinding. Responders were offered the option to remain on medication.

Trial 2: Concurrent treatment with Prolonged Exposure (COPE) is a 12-week intervention that integrates the empirically-supported models of prolonged exposure for PTSD [44] and RPT for SUD [45,46]. Participants had up to 14 weeks for treatment completion. Sessions 1 to 3 focused on goal-setting, psychoeducation and cognitive-behavioral strategies. To address behavioral avoidance and fear associated with trauma memories, in vivo and imaginal exposures began in Sessions 4 and 5, respectively, and continued until Session 11. Relapse prevention strategies were integrated with the prolonged exposure sessions during each 90-min session. Participants recorded progress of exposure exercises, substance use cravings and use of coping skills. Relapse Prevention Therapy (RPT) [45,46] is a cognitive-behavioral SUD intervention that focuses on coping strategies to effectively manage situations that increase the risk of relapse. Psychoeducation, role-playing and active problem-solving exercises are combined with at-home assignments and geared towards increasing participants’ self-efficacy in preventing relapse.

### 2.3. Measures

Demographics: In both trials: age, sex, race/ethnicity, education, marital status, employment pattern and income were collected during the baseline interview.

Psychiatric and alcohol/substance use disorder diagnoses: In both trials, the Structured Clinical Interview for DSM-IV for Axis I Disorders (SCID-I) [47] was administered at baseline and follow-ups to assess current Alcohol Use Disorder (AUD) and Substance Use Disorder (SUD) diagnoses, age of AUD/SUD onset and the presence of any other current or past anxiety, mood or psychotic disorders. Axis II (personality) disorders were not assessed. AUD/SUD diagnoses were considered current if diagnostic criteria were met in the prior six months. The SCID-I has demonstrated high interrater reliability [47].

PTSD symptom severity: In both trials, the Clinician-Administered PTSD Scale (CAPS) [48] was used at baseline and follow-ups to measure symptom severity in the previous 30 days. The scale consists of the Re-Experiencing, Avoidance/Numbing and Hyperarousal symptom cluster subscales. The frequency and intensity scores for each symptom cluster subscale are summed to obtain an overall total scale score. The CAPS total scores ranges from 0 to 136. Higher scores indicate greater severity. Clinical assessors received formal training in administering the CAPS. The Modified PTSD Symptom Scale Self-Report (MPSS-SR) [49] was used weekly during treatment to assess self-reported symptom severity in the previous 7 days. Although the instruments have different ranges, both yield a total score comprised of the sum of frequency and intensity ratings of each of the 17 DSM-IV-TR PTSD symptoms. Psychometric studies of the MPSS-SR with similar comorbid PTSD + SUD treatment samples demonstrated its high concurrent validity with the CAPS and suggest that it is a reliable tool for monitoring PTSD symptoms [50].

Substance use: In Trial 1, The Timeline Follow-Back (TLFB) [51] was used to assess alcohol use patterns at baseline, weekly during the trial and all follow-ups. Participants retrospectively estimated their daily alcohol consumption in the previous 30 days with a detailed calendar to help orient them toward patterns in their drinking and specific episodes of erratic or binge drinking. TLFB has demonstrated good reliability as an instrument for the estimation of daily alcohol consumption [52].

In Trial 2, primary SUD diagnosis was based on a number of dependence criteria from the SCID-I. The Addiction Severity Index-Lite (ASI-Lite) [53,54], a semi-structured clinical interview, was used to assess the frequency of primary substance use in the previous 30 days at baseline and follow-ups. The ASI-lite had demonstrated good reliability and validity as a measure of the frequency of substance use and associated consequences [54].

In both trials, the Substance Use Inventory (SUI) [55] was used to assess self-reported frequency of days of cannabis use in the previous 7 days at baseline, weekly during the trial and all follow-ups.

### 2.4. Statistical Analyses

Chi-square tests for categorical variables and independent sample *t*-tests for continuous variables were used to compare cannabis users and nonusers on baseline sociodemographic variables. Bivariate correlations were utilized to assess the associations between baseline frequency of cannabis use, age, end-of-treatment PTSD symptom severity and days of primary substance use. A series of multivariate regression analyses was conducted to determine whether frequency of marijuana use at baseline predicted end-of-treatment response on two dimensions (PTSD symptom severity (as measured by the CAPS) and days of primary substance use (as measured by ASI and TLFB)). End-of-treatment PTSD symptom severity and days of primary substance use were regressed independently on the frequency of marijuana use while controlling for baseline scores of either PTSD symptom severity or days of primary substance use, gender and age. All analyses were run using bootstrap sampling (1000 bootstrap draws) in order to reduce the chances of a confirmation of null findings due to Type I error related to sample size.

Several models were specified in a generalized multilevel framework using Stata Version 14 [56] to examine the cross-lagged impact of cannabis use and primary substance use, as well as the impact of lagged PTSD symptom severity and the impact of lagged cannabis and primary substance use on PTSD symptom severity. These models take advantage of the weekly treatment data, while accounting for nesting (time nested within individuals). For the cross-lagged model, a two-level, multivariate model was specified with the following Equation (1):

(1) Yijd=(exp(β0ia+β1ia(week−2)ij+β2ia(cannabis use)ij−1Yijd=(exp(β0ia+β3ia(cannabis use)ij−1 x(week−2)ij+β2ia(PTSD)ij−1Yijd=(exp(β0ia+β2ia(PTSD)ij−1 x(week−2)ij+εij))+(exp(β0ibYijd=(exp(β0ia+β1ib(week−2)ij+β2ib(primary substance use)ij−1Yijd=(exp(β0ia+β3ia(primary substance use)ij−1 x(week−2)ij+β2ia(PTSD)ij−1Yijd=(exp(β0ia+β2ia(PTSD)ij−1 x(week−2)ij+εij))

In this equation, the information to the left of the bolded plus sign represents our first dependent variable, primary substance use, which is denoted by the subscript *a*, while the information to the right of the plus sign represents our second dependent variable, cannabis use, which is denoted by the subscript *b*. To model the impact of lagged variables (i.e., weekly cannabis use, weekly primary substance use, weekly PTSD symptom severity), the variables were lagged by one time point, which is seen in the above equation by the subscript *j* − 1. Given that both cannabis use and primary substance use were lagged in the model, time itself was centered at Week 2 of treatment so that the predicted intercept (i.e., when *x* = 0) for each variable would be meaningful.

When we were examining the impact of lagged weekly cannabis and primary substance use on PTSD symptoms, we were unable to model all of the variables as outcomes simultaneously, as we did for primary substance use and cannabis use, because of the difference in scales of the substance use variables and the PTSD symptom severity [57]. Therefore, a two-level, univariate model was specified in this case with the following Equation (2):

(2) PTSDij=(exp(β0ia+β1ia(week−2)ij+β2ia(cannabis use)ij−1PTSDij=(exp(+β3ia(cannabis use)ij−1 x(week−2)ijPTSDij=(exp(+β2ib(primary substance use)ij−1PTSDij=(exp(+β3ia(primary substance use)ij−1 x(week−2)ij

Given that both primary substance use and cannabis use were significantly right skewed and there was evidence of overdispersion, a negative binomial family distribution and a log link function were used for all models in which substance use was the outcome. When PTSD symptom severity was the outcome, a normal distribution was specified. For both models, the unadjusted model controlled only for time, while sex and age (mean centered) were included as covariates in adjusted models. To present the most parsimonious model, the final model presented includes only significant predictors.

## 3. Results

Table 2 shows the zero-order correlations between baseline days of cannabis use and sociodemographic and outcome variables. The frequency of cannabis use was significantly positively correlated with days of urge, craving or desire to use cannabis (*r* = 0.84, *p* < 0.01). There were no significant correlations between baseline days of cannabis use and end-of-treatment PTSD symptom severity or days of primary substance use.

Multivariate regressions with bootstrapping further revealed that baseline days of cannabis use was not a significant predictor of end of treatment PTSD symptom severity (β = 0.11; *t* = 1.04, *p* = 0.30) or end-of-treatment days of primary substance use (β = 0.25; *t* = 0.20, *p* = 0.84), indicating that cannabis use was not associated with either positive or negative response at end-of-treatment in either domain. Neither gender, nor age accounted for a significant amount of variance in end-of-treatment PTSD symptom severity or days of primary substance use (*p* > 0.05). Only baseline PTSD symptom severity accounted for a significant proportion of variance in the end-of-treatment PTSD symptom severity (β = 0.35; *t* = 3.58, *p* < 0.01). The overall model fit for predicting end-of-treatment PTSD symptom severity was *R*^2^ = 0.14 and for end-of-treatment days of primary substance use was *R*^2^ = 0.04.

A multilevel linear growth model was fit to the data to examine the cross-lagged associations between weekly cannabis and primary substance use throughout the 12 weeks of treatment. Although both outcomes were run simultaneously, both unadjusted and adjusted model results are displayed in two tables (Table 3 and Table 4) to increase clarity. In the unadjusted model, results indicated that individual’s weekly primary substance use decreased significantly during treatment (incident rate ratio (IRR) = 0.91, 95% CI (0.87, 0.95), *p* < 0.001), while there was a trend for a decrease in weekly cannabis use (IRR = 0.90, 95% CI (0.81, 1.00), *p* = 0.071). There was evidence of a cross-lagged association for both weekly cannabis (IRR = 0.74, 95% CI (0.67, 0.80), *p* < 0.001) and primary substance use (IRR = 0.83, 95% CI (0.74, 0.93), *p* = 0.002), both indicating that as one substance increased, the other substance decreased. Lagged PTSD symptom severity was not significantly associated with either weekly primary substance or cannabis use (*p* = 0.337 and 0.746, respectively) and none of the lagged predictors (i.e., weekly cannabis use, weekly primary substance use, PTSD symptom severity) had a significant interaction with time. After adjusting for age and sex, results indicated that both weekly primary substance use (IRR = 0.91, 95% CI (0.89, 0.93), *p* < 0.001) and weekly cannabis use (IRR = 0.91, 95% CI (0.86, 0.96), *p* < 0.001) significantly decreased during treatment. Further, the cross-lagged associations between weekly cannabis use and weekly primary substance use were largely unchanged. Specifically, a one-unit increase in lagged weekly cannabis use was associated with 26% lower weekly primary substance use, while a one-unit increase in lagged primary substance use was associated with 14% lower weekly cannabis use.

**Figure 1:**
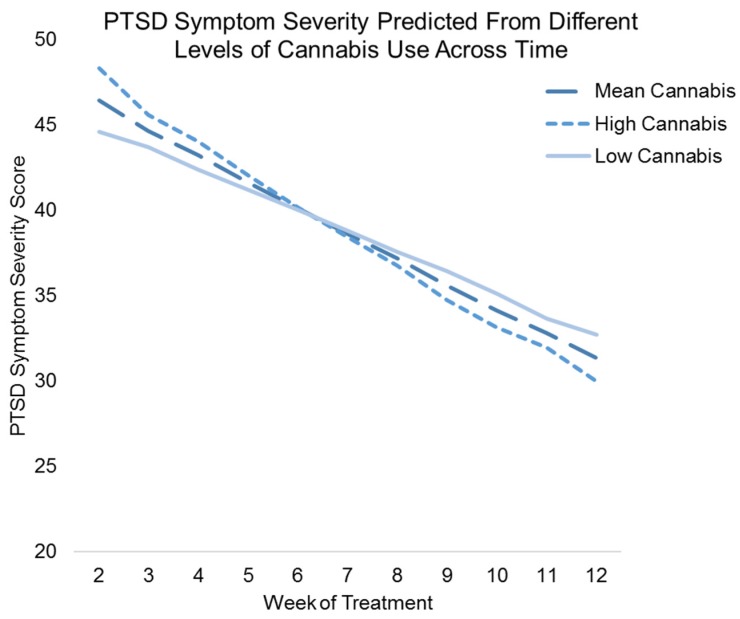
Figure showing the adjusted results of weekly lagged cannabis use and weekly lagged cannabis use *x* time, predicting weekly PTSD symptom severity scores. The *x*-axis represents the time from week 2 of treatment to week 12 of treatment (week 1 is not represented because the independent variable is lagged one time-point), while the *y*-axis represents the predicted score on the PTSD symptom severity measure. Results show a crossover effect, whereby higher weekly lagged cannabis use was associated with greater weekly PTSD symptom severity early in treatment, but lower weekly PTSD symptom severity later in treatment.

A second multilevel linear growth model was fit to the data to examine the associations between lagged weekly cannabis and primary substance use on PTSD symptom severity throughout the 12 weeks of treatment. Both unadjusted and adjusted results are displayed in Table 5. In the unadjusted results (β = −1.60, 95% CI (−2.06, −1.13), *p* < 0.001), PTSD symptom severity decreased significantly during treatment. However, neither lagged weekly cannabis, nor primary substance use was associated with weekly PTSD symptom severity (*ps* = 0.128 and 0.901, respectively). In addition, lagged weekly primary substance use did not interact with time in the prediction of PTSD symptom severity scores (*p* = 0.902). However, the interaction between lagged weekly cannabis use and time was significant (β = −0.23, 95% CI (−0.43, −0.02), *p* = 0.029). After adjusting for covariates, results remained largely unchanged. The interaction between lagged weekly cannabis use and time was explored (see Figure 1) and indicated that there was a crossover effect; although higher weekly lagged cannabis use was associated with greater PTSD symptom severity early in treatment, later in treatment, higher weekly lagged cannabis use was associated with lower PTSD symptom severity.

## 4. Discussion

This study examined the associations between cannabis use and in-treatment and end-of-treatment PTSD symptom severity and frequency of primary substance use among a sample of adults with co-occurring PTSD and substance use disorders who received cognitive-behavioral therapy. Approximately 24% of the sample reported cannabis use prior to treatment, and more men were cannabis users than non-users. Cannabis users were more likely to have comorbid cocaine use disorders, whereas nonusers were more likely to have an alcohol use disorder. This finding is consistent with prior studies showing that approximately 60% to 90% of those with cocaine use disorder also use cannabis [58]. Analyses revealed that baseline cannabis use was neither negatively, nor positively associated with end-of-treatment PTSD symptom severity. In contrast to a line of studies showing that individuals with PTSD, particularly those who struggle with hyperarousal and sleep problems, may be more likely to use cannabis to cope with their traumatic stress symptoms [19,59], cannabis use was independent of symptom outcomes at end-of-treatment across participants undergoing cognitive behavioral therapy for PTSD and SUD. Our results also revealed a cross-over effect when examining the lagged association between weekly cannabis use and weekly PTSD symptoms during 12 weeks of treatment. Higher cannabis use was associated with higher PTSD symptom severity early in treatment, but lower PTSD symptom severity later in treatment. It is possible that early in treatment, cannabis may be used frequently in order to ameliorate high levels of PTSD symptoms. However, later in treatment, high cannabis use may interact synergistically with psychological treatment to reduce PTSD symptoms. The present study, however, was not able to examine the interaction between cannabis use and treatment; thus, these hypotheses are speculative. Future studies are recommended to replicate and tease apart the mechanisms of these findings.

The lack of association between baseline cannabis use and end-of-treatment frequency of primary substance use is consistent with a set of research suggesting cannabis use is independent of other substance use, particularly after treatment [33,34,35,36]. Importantly, findings from the present study further suggest that cannabis use before treatment is not associated with adverse PTSD outcomes in this sample. One of the clinically-salient questions in treatment of concurrent substance use involves what position clinicians should take regarding concurrent use of cannabis among other illicit substance users. Abstinence model perspectives would argue that any use of any substance type in ongoing treatment would be discouraged. In contrast, harm reduction models would identify reductions in drug use severity measured in metrics involving both type of substance used (i.e., more or less public health impact) and also the amount of use by type of substance as key indicators of treatment improvement [60]. Findings from our cross-lagged analyses bear on these perspectives. Analyses revealed that as weekly cannabis use increased, subsequent weekly primary substance use decreased and vice versa. These findings contrast with studies showing that ongoing regular cannabis use was a significant predictor of subsequent increases in illicit drug use [23]. It is possible that, in this sample, the psychoactive properties of cannabis reduce the likelihood that participants will utilize their primary substance of choice. Alternatively, increases in weekly primary drug of choice may decrease the desire for cannabis. Regardless, from a harm reduction perspective, the utilization of cannabis instead of other illicit substances and alcohol may be associated with less harmful consequences and, thus, may be seen as a more acceptable intermediate outcome.

Several limitations should be noted. The lack of association between baseline cannabis use and end-of-treatment outcomes should be interpreted with caution given the small sample size of cannabis users. Moreover, we utilized frequency/days of cannabis use instead of quantity or severity of use, which could have been more sensitive to detecting associations among these variables. Further, because we utilized the global category of primary substance use, we were not able to tease apart the specific associations between cannabis and specific types of drugs (e.g., cocaine, alcohol or heroin). Self-report measures of substance use bring inherent biases related to recall and social desirability. Finally, since the study was not originally designed to test the impact of cannabis use on treatment outcomes, these post-hoc subgroup analyses may suffer from selection bias.

Despite these limitations, this is one of the first studies to examine the impact of cannabis use in a racially-/ethnically-diverse sample of participants receiving cognitive behavioral therapies for co-occurring PTSD and substance use disorders. In addition to documenting moderate rates of cannabis use among those with other primary substance use disorders, these findings provide evidence in support of a harm reduction perspective in the treatment of addictions with PTSD. First, our findings demonstrated that baseline cannabis use was not associated with end-of-treatment outcomes, suggesting independent pathways of change. Second, cross-lagged predictive models examining the impact of previous week cannabis use on subsequent primary substance use revealed an inverse relationship showing that increasing cannabis use was actually associated with lowering primary substance use. Conversely, increasing primary substance use in the previous week was associated with lowering cannabis use the following week. Moreover, findings revealed a cross-over effect, whereby higher cannabis use was related to higher PTSD symptom severity early in treatment, but lower PTSD symptom severity later in treatment. Taken as a whole, our findings provide more support in the controversy that cannabis use (in non-primary cannabis dependence users) is not necessarily an adverse component to the treatment process for addictions.

## Figures and Tables

**Table 1:** Participants’ characteristics by cannabis use. SUD, Substance Use Disorder.

Variables		Non-Users (*n* = 104)	Cannabis Users (*n* = 32)	Statistics
		M (SD) or %		
Age		44 (9.18)	41.63 (9.38)	*t* = 1.273, *p* = 0.205
Gender (Male %)		42% (*n* = 44)	66% (*n* = 21)	*χ*^2^ = 5.33, *p* = 0.021
Education		13.28 (2.52)	13.08 (1.88)	*t* = 0.426, *p* = 0.671
Employment in the Past 3 Years *	% Employed	69.23% (*n* = 72)	78.13% (*n* = 25)	*χ*^2^ = 5.12, *p* = 0.402
	% Unemployed	30.77% (*n* = 32)	21.87% (*n* = 7)	
Race/Ethnicity ^†^	% White	21.87 (*n* = 21)	12.5 (*n* = 4)	*χ*^2^ = 5.12, *p* = 0.163
	% Black	59.38 (*n* = 57)	56.25 (*n* = 18)	
	% Hispanic	12.5 (*n* = 12)	28.12 (*n* = 9)	
	% Other (i.e., Native American)	6.25 (*n* = 6)	3.125 (*n* = 1)	
Cocaine Use Disorder (% Yes) ^†^		37.5 (*n* = 36)	59.37 (*n* = 19)	*χ*^2^ = 4.686, *p* = 0.030
Alcohol Use Disorder (% Yes) ^†^		94.79 (*n* = 91)	78.13 (*n* = 25)	*χ*^2^ = 7.847, *p* = 0.005
Other SUD (Opioid, Hallucinogen; % Yes) ^†^		5.2 (*n* = 5)	6.25 (*n* = 2)	*χ*^2^ = 3.403, *p* = 0.334

* Employed included people that reported working full time or part time. Unemployed included people who reported being students, retired or unemployed. ^†^ The non-user group had missing data for 8 people. The results are reported based on a sample of 96 people for the non-user group.

**Table 2:** Correlations between study variables.

Variables	Age	Education	Days of Cannabis Use ^†^	Days of Urge, Desire or Craving for Cannabis ^†^	Baseline Primary Substance Use ^‡^	End-of-Treatment Primary Substance Use ^‡^	Baseline CAPS ^1^	End-of-Treatment CAPS ^1^
Age	1	0.101	−0.009	−0.031	−0.011	−0.077	0.006	−0.051
Education		1	−0.114	−0.194	−0.091	0.129	−0.022	−0.001
Days of cannabis use ^†^			1	0.844 **	0.007	0.004	0.069	0.157
Days of urge, desire or craving for cannabis ^†^				1	0.073	−0.028	0.113	0.081
Baseline Primary Substance use **^‡^**					1	0.049	0.094	−0.016
End-of-treatment Primary Substance use **^‡^**						1	0.140	0.370 **
Baseline CAPS							1	0.377 **
End-of-treatment CAPS								1

^1^ CAPS = Clinician-Administered PTSD Scale; ** *p* < 0.01, results are based on 1000 bootstrap samples; **^‡^** number of days of primary substance use in past 30 days; ^†^ number of days of use in the past 7 days.

**Table 3:** Exponentiated regression coefficients for models examining the association of cross-lagged weekly cannabis use on primary substance use.

Predictors	Unadjusted Model IRR (95% CI)	Final Adjusted Model IRR (95% CI)
Status at Week 2 of Treatment:		
Intercept	7.81 (5.05, 12.07) ***	7.12 (5.32, 9.53) ***
Sex (female)	--	NS
Age	--	0.96 (0.93, 0.98) ***
Lagged Cannabis Use	0.74 (0.67, 0.80) ***	0.74 (0.69, 0.79) ***
Lagged PTSD	1.00 (1.00, 1.01)	NS
Rate of Change:		
Time (linear)	0.91 (0.87, 0.95) ***	0.91 (0.89, 0.93)
Lagged Cannabis Use *X* Time (linear)	0.98 (0.96, 1.00)	NS
Lagged PTSD *X* Time (linear)	1.00 (1.00, 1.00)	NS

Note: This model was specified with a negative binomial distribution. Negative binomial regression coefficients are expected differences in log counts. All coefficients presented here are exponentiated. When exponentiated, the intercept represents an expected count of days of primary substance use (when time = 0), and the slope represents a ratio of expected counts or an incident rate ratio. All predictors are also interpreted as ratios of expected counts or incident rate ratios (IRRs). For example, the IRR of 0.74 for lagged cannabis use is interpreted to mean that a one-unit change in lagged cannabis use was associated with a 26% lower expected count of primary substance use (when all other predictors were held constant). Sex was coded dichotomously with males equal to 0, while age was mean centered. *** *p* < 0.001.

**Table 4:** Exponentiated regression coefficients for models examining the association of cross-lagged primary substance use on weekly cannabis use.

Variables	Unadjusted Model IRR (95% CI)	Final Adjusted Model IRR (95% CI)
Status at Week 2 of Treatment:		
Intercept	1.71 (0.85, 3.45)	2.25 (1.42, 3.53) ***
Sex (female)	--	0.23 (0.15, 0.34) ***
Age	--	0.92 (0.89, 0.95) ***
Lagged Primary Substance Use	0.83 (0.74, 0.93) **	0.86 (0.80, 0.93) ***
Lagged PTSD	1.00 (1.00, 1.01)	NS
Rate of Change:		
Time (linear)	0.90 (0.81, 1.00)	0.91 (0.86, 0.96) ***
Lagged Primary Substance use *X* Time (linear)	1.00 (0.97, 1.02)	NS
Lagged PTSD *X* Time	1.00 (1.00, 1.00)	NS

Note: This model was specified with a negative binomial distribution. Negative binomial regression coefficients are expected differences in log counts. All coefficients presented here are exponentiated. When exponentiated, the intercept represents an expected count of days of cannabis use (when time = 0), and the slope represents a ratio of expected counts or an incident rate ratio. All predictors are also interpreted as ratios of expected counts or incident rate ratios (IRRs). For example, the IRR of 0.86 for lagged primary substance use is interpreted to mean that a one-unit change in lagged primary substance use was associated with a 14% lower expected count of cannabis use (when all other predictors were held constant). Sex was coded dichotomously with males equal to 0, while age was mean centered. ** *p* < 0.01; *** *p* < 0.001.

**Table 5:** Exponentiated regression coefficients for models examining the impact of lagged weekly cannabis use and primary substance use on PTSD symptom severity.

Variables	Unadjusted Model	Adjusted Model
Status at Week 2 of Treatment:		
Intercept	45.41 (39.85, 50.98) ***	45.71 (40.65, 50.77) ***
Lagged Cannabis Use	1.04 (−0.30, 2.37)	NS
Lagged Primary Substance Use	−0.06 (−1.05, 0.93)	0.98 (−0.25, 2.21)
Sex (female)	--	NS
Age	--	NS
Rate of Change:		
Time (linear)	−1.60 (−2.06, −1.13) ***	−1.41 (−1.74, −1.07) ***
Lagged Cannabis Use *X* Time (linear)	−0.23 (−0.43, −0.02) *	−0.23 (−0.44, −0.03) *
Lagged Primary Substance Use *X* Time (linear)	0.01 (−0.15, 0.17)	NS

Note: This model was specified with a normal distribution. The intercept represents the expected value of PTSD symptom severity when all other predictors are held constant at 0, while the slope represents the expected difference in PTSD symptom severity given a one-unit change in time (all other variables held constant). All predictors are interpreted similarly to the slope and represent the expected difference due to a one-unit change in the predictor (all other predictors held constant). Sex was coded dichotomously with males equal to 0, while age was mean centered. * *p* < 0.05; *** *p* < 0.001.
